# Limited stage high grade B-cell lymphoma with *MYC, BCL2* and/or *BCL6* rearrangements*: BCL2* rearrangements drives the poor outcomes

**DOI:** 10.1038/s41408-024-01148-x

**Published:** 2024-10-14

**Authors:** Jennifer K. Lue, Efrat Luttwak, Alfredo Rivas-Delgado, Helen Irawan, Alexander Boardman, Philip C. Caron, Kevin David, Zachary Epstein-Peterson, Lorenzo Falchi, Paola Ghione, Paul Hamlin, Steven M. Horwitz, Andrew M. Intlekofer, William Johnson, Anita Kumar, Alison Moskowitz, Ariela Noy, M. Lia Palomba, Ralphael Steiner, Robert Stuver, Pallawi Torka, Santosha Vardhana, Andrew D. Zelenetz, Heiko Schoder, Brandon Imber, Joachim Yahalom, Yanming Zhang, Pallavi Galera, Ahmet Dogan, Umut Aypar, Gilles Salles

**Affiliations:** 1 Lymphoma Service, Division of Hematological Malignancies, Department of Medicine, Memorial Sloan Kettering Cancer Center, New York, NY USA; 2 Department of Pathology and Laboratory Medicine, Memorial Sloan Kettering Cancer Center, New York, NY USA; 3 Department of Radiology, Memorial Sloan Kettering Cancer Center, New York, NY USA; 4 Department of Radiation Oncology, Memorial Sloan Kettering Cancer Center, New York, NY USA

**Keywords:** B-cell lymphoma, B-cell lymphoma, Prognosis

Dear Editor,

High-grade B-cell lymphoma with *MYC, BCL2* and/or *BCL6* rearrangements (HGBCL-DH/TH*)* is a group of aggressive lymphomas that are associated with poor outcomes when treated with R-CHOP [1, 2]. Conflicting data exists regarding the outcomes of HGBCL with *MYC and BCL2* rearrangement*s* (DH-*BCL2*) as compared to HGBCL with *MYC and BCL6* translocations (DH-*BCL6*) [3–6]. The International Consensus Classification of Mature Lymphoid Neoplasms has proposed 2 subtypes of HGBCL-DH: HGBCL with *MYC* and *BCL2* rearrangements (with/without *BCL6 r*earrangement), and HGBCL with *MYC* and *BCL6* rearrangements (R) [7]. The Fifth Edition of the WHO Classification of Haematolymphoid Tumors has re-classified DH-*BCL6* into DLBCL-NOS or HGBCL-NOS given their unclear poor outcomes and lack of common genetic pattern [8]. Supporting this, when analyzed with the LymphGen classification system [9] or the Cluster classification [10], DH-*BCL2* aligns with EZB/C3 cluster and is often enriched in *TP53* mutations, whereas DH-*BCL6* is either EZB or BN2 subtypes, and typically lacks *TP53* aberrancy [11]. HGBCL-TH are best characterized as EZB and *BCL6-R* is considered a late event [11].

There is a paucity of data regarding the outcomes and biological factors in limited-stage (LS) HGBCL-DH/TH, as studies are dominated by Stage III/IV patients. In a retrospective study of LS aggressive B-cell lymphomas with *MYC*-R, HGBCL-DH/TH patients experienced similar progression-free survival (PFS) and overall survival (OS) when treated with intensive chemotherapy versus R-CHOP [12]. The role of *MYC* translocation partner (Immunoglobulin (Ig) vs. non-Ig) has been correlated with clinical outcomes in DH/TH, however, it has not been fully explored in LS [3].

We performed an IRB approved retrospective analysis of patients with newly diagnosed HGBCL-DH/TH at Memorial Sloan Kettering Cancer Center from January 1, 2010, to September 30, 2022. Stage modified IPI (smIPI), Stage Adjusted IPI (stIPI), and CNS International Prognostic Index (CNS-IPI) were calculated (Table 1 legend). PFS and OS were estimated using the Kaplan-Meier method; comparisons were made using log-rank test. Associations between clinical outcomes and treatment characteristics were evaluated using Cox regression models. Statistical programs included SPSS software (IBM SPSS Statistics for Windows, version 25) and R (R version 3.6.3). FISH was performed using the following probes: *IGH* (Abbott Molecular), I*GK* (Cytocell), *IGL* (Cytocell) break-apart probes and/or MYC-IGH (Abbott Molecular), MYC-IGK (Cytotest), MYC-IGL (Cytotest) dual-fusion probes. One hundred interphase cells were analyzed on archived FFPE.

**Table 1:** Baseline Characteristics.

	All Patients	HGBCL *MYC/BCL2*
	*N* = 41	*N* = 32
Median Age (range, Q1, Q3)	59.9 (22.3–87.7; Q1 53.0, Q3, 73.2)	57.9 (22.3–87.7; 53.0, 73.2)
Sex (*n*,%)		
Male	24 (59%)	18 (56%)
Female	17 (41%)	14 (44%)
Ann Arbor Stage		
Stage I	15 (37%)	13 (41%)
Stage II	26 (63%)	19 (59%)
Extranodal Involvement	14 (34%)	11 (34%)
Baseline Bone Marrow Biopsy	23 (56%)	18 (56%)
Bulky (≥ 7 cm)	16 (44%)	12 (37%)
ECOG		
0–1	16 (40%)	18 (42%)
≥ 2	24 (60%)	13 (58%)
LDH		
Normal	19 (61%)	16 (70%)
Elevated	12 (39%)	7 (30%)
smIPI^a^		
0–1	21 (65%)	16 (67%)
2–3	11 (35%)	8 (33%)
stIPI^b^		
0–1	24 (75%)	18 (75%)
2–3	8 (25%)	6 (25%)
Transformed	10 (24%)	8 (25%)
Cell of Origin^c^		
Germinal Center	39 (97.5%)	30 (97%)
Non-Germinal Center	1 (2.5%)	1 (3%)
Translocations		
*MYC/BCL2*	17 (41%)	17 (53%)
*MYC/BCL6*	9 (22%)	−
*MYC/BCL2/BCL6*	15 (37%)	15 (47%)
Median Lines of Therapy^d^	1 (1–5)	1 (1–5)
Previously untreated	38 (93%)	30 (94%)
Previously treated for iNHL	3 (7%)	2 (6%)
Diagnosis to Treatment Interval	23 days	21.5 days
First Line Treatment		
DA-R-EPOCH	31 (76%)	24 (75%)
R-CHOP	8 (20%)	6 (19%)
Other^e^	2 (4%)	2 (6%)
Median # Cycles (range)	6 (1–8)	6 (1–8)
Consolidative Radiation	10 (24%)	7 (23%)

^a^Stage modified IPI (smIPI) [age>60, elevated LDH, Stage II/IIE, ECOG performance status greater than/or equal to 2].

^b^Stage Adjusted IPI (stIPI) [age >60, elevated LDH, ECOG performance status greater than/equal to 2].

^c^Cell of origin determined by Han’s Algorithm. There was insufficient immunohistochemistry data for one patient with TH.

^d^Median lines of therapy for treatment of HGBCL with MYC/BCL and/or BCL6. Of the patients with transformation, 3 patients received prior therapy for an indolent lymphoma [radiation *n* = 1, R-CVP (*n* = 1), Rituximab-Bendamustine (*n* = 1)].

^e^Other: R-GCVP(*n* = 1), R-CODOX-M-IVAC (*n* = 1).

Q1=quartile 1; Q3=quartile 3.

One hundred and eighty-eight patients were identified with HGBCL-DH/TH. Nine subjects were excluded due to limited information. Forty-one patients had stage I/II disease. Baseline characteristics are described in Table 1. Forty-one percent of patients had DH*-BCL2* (*n* = 17), 22% had DH-*BCL6* (*n* = 9) and 37% had HGBCL-TH (*n* = 15). The median DTI was 23 days. DA-R-EPOCH (76%) was the most common therapy. Eleven patients (24%) received consolidative radiation.

The overall response rate (ORR) and complete response rate to first-line chemoimmunotherapy were both 73%. After a median follow-up of 48 months, the median PFS was not reached, and OS was 63 months (Fig. 1A, B). The 2-year PFS and OS was 55% and 67%, respectively. Fifteen patients had relapse/refractory (R/R) disease; 12 were primary refractory. There was no difference in DTI between primary refractory patients (median DTI 19) versus non-refractory patients (median DTI 23 days) (*p* = 0.97). Seven patients received salvage therapy, including autologous stem cell transplant (ASCT) (*n* = 1), chimeric antigen receptor (CAR) T-cell therapy (*n* = 3), or both (*n* = 3). Most of the patients received CAR T-cell therapy in ≥3 line. Twelve of the 15 R/R patients died due to progressive disease. At censorship, among the 3 survivors, 2 patients received CAR T-cell therapy with one previously undergoing ASCT and the other received CAR-T in the second-line; the third patient received rituximab-bendamustine-polatuzumab vedotin complicated by acute myeloid leukemia.

**Fig. 1:**
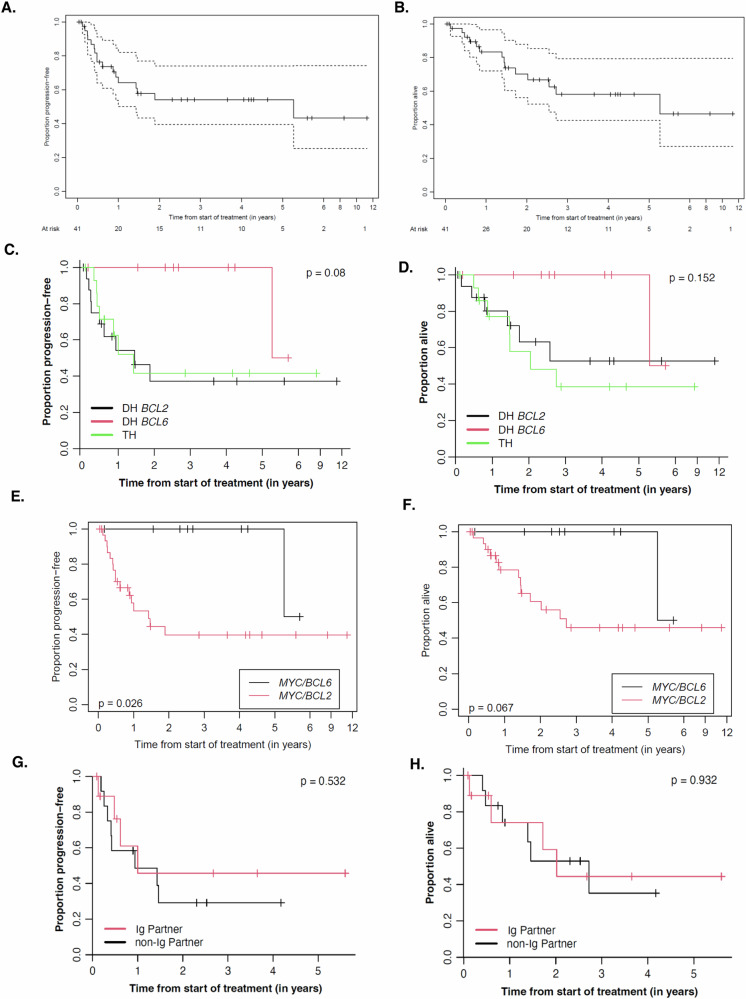
Outcomes in Patients with LS HGBCL-DH/TH Patients. **A**, **B** PFS and OS, respectively, for the entire cohort. Dash lines represent confidence bands. **C** PFS of DH-*BCL2*, DH-*BCL6* and TH. No difference in overall PFS when comparing the three groups (*p* = 0.08). 2-year PFS of DH-*BCL2* group was 37% (95% CI 18.3–75.5); 2-year PFS of DH-*BCL6* 100%; *p* = 0.03. **D** OS of DH-*BCL2*, DH-*BCL6* and TH. No difference in overall OS when comparing three groups (*p* = 0.152). 2-year OS of DH-BCL2 was 63% (95% CI 42–96%); 2-year OS of DH-BCL6 was 100%; *p* = 0.1. **E** Inferior PFS demonstrated in patients with *MYC/BCL2* rearrangements (DH-*BCL2* and TH) versus *MYC/BCL6* rearranged patients. **F** No impact on OS when comparing *MYC/BCL2* rearranged patients (DH-*BCL2* and TH) versus *MYC/BCL6* rearranged patients. **G** PFS assessment between *MYC*-Ig partner versus *MYC-*non-Ig partner. **H** OS assessment between *MYC*-Ig partner versus *MYC-*non-Ig partner.

DH-*BCL2* displayed an inferior 2-year PFS of 37% (95% CI 18.3–75.5), as compared to 100% in DH-*BCL6* (*p* = 0.03) (Fig. 1C). There was no additional adverse impact on 2-year PFS between TH (2-year PFS 41.7%, 95% CI 20.6–84.4) as compared to DH-*BCL2* (*p* = 0.8). The 2-year OS for patients with DH-*BCL2* was 63% (95% CI 42–96%) as compared to 100% for DH-*BCL6* (Fig. 1D). The 2-year PFS and OS for the *MYC/BCL2-R* [DH-BCL2 + TH] group were 39.6% and 60.7%. The presence of *MYC*/*BCL2-R* led to an inferior PFS (*p* = 0.026) as compared to *MYC/BCL6-R*; a similar trend was observed for OS (*p* = 0.067) (Fig. 1E, F). The only death in the DH-*BCL6* group was unrelated to lymphoma, whereas all deaths in the *MYC/BCL2-R* cohort were due to disease progression (*n* = 12).

In a univariate analysis, elevated LDH (*p* = 0.02; *p* = 0.03), smIPI score ≥2 (*p* = 0.01; *p* = 0.02) and stIPI score ≥2 (*p* = 0.01; *p* = 0.001) were associated with statically significant inferior PFS and OS, respectively. Extranodal involvement, bulky disease, transformed indolent lymphoma, P53 overexpression, intensive chemotherapy regimen or radiation had no impact on PFS. Baseline ECOG, extranodal involvement, smIPI, stIPI, bulky disease, or elevated LDH did not differ between patients who received intensive chemotherapy compared to non-intensive chemotherapy. Thirty-one subjects had archival tissue available for *MYC* partner analysis which was completed in 22 patients. Ig vs. non-Ig *MYC* partner had no impact on PFS (*p* = 0.53) or OS (*p* = 0.93) (Fig. 1G, H).

In this cohort, 68.2% (*n* = 28) of patients had a low CNS-IPI score (0–1), 29.4% (*n* = 12) with an intermediate risk score [2, 3], while 2.4% (*n* = 1) had a high-risk score [4–6]. Thirty-two percent (13/41) of patients received CNS prophylaxis, with the majority receiving intrathecal chemotherapy (median=4). No CNS relapses were observed.

Our study demonstrates that *MYC/BCL2*-*R* are associated with poor outcomes compared to *MYC*/*BCL6*-*R*. This data adds to the growing literature indicating DH-*BCL6* is not an adverse prognostic factor and it is a separate entity from DH-*BCL2* and HGBCL-TH. It also supports managing DH-*BCL6* with less intensive regimens. With the poor outcomes reported for DH-*BCL2* and TH patients, the additive use of consolidative radiation is unclear. Our data supports a prior study [12] where there was no impact of consolidative radiation on both PFS and OS in LS *MYC* rearranged lymphomas.

Recent analyses have revealed that a common gene signature, the ‘Dark Zone Signature’ (DZSig), is present in DH-*BCL2* and other germinal center (GC) DLBCLs, and is associated with inferior OS compared to GC-DLBCLs without DZSig [6]. The role of DZSig in LS HGBCL-DH/TH is unclear given the small numbers of Stage I/II patients. However, there was a non-statistically significant shorter 2-year freedom from progression and 2-year OS of 77% and 85%, respectively, in the LS DLBCL patients harboring the DZSig as compared to LS GCB-DLBCL (94% and 92%), and LS ABC-DLBCL (86% and 85%) [6]. An inferior 2-year OS was observed in patients with LS DH-*BCL2* (75%) as compared to LS Non-HGBCL-DH (89%) (*p* < 0.05) after treatment with R-CHOP irrespective of presence of DZSig. We observed a similar suboptimal 2-year OS in our LS DH-*BCL2* cohort in patients primarily treated with DA-R-EPOCH. Therefore, renewed efforts focusing on optimizing front-line therapy for these high-risk patients are necessary. The role of polatuzumab-vedotin (Pola) in combination with R-CHP for the treatment of LS HGBCL-DH/TH is unclear, as there were limited patients fitting this criteria in the study; notably there was no improvement in HGBCL-DH/TH patients [13]. However, in a separate post-hoc analysis, there was a beneficial effect of Pola-R-CHP in patients with EZB and DZSig, which may lend itself to potential treatment implications in patients with LS HGBCL DH/TH [14]. Dedicated studies are required to reconcile these outcomes.

Primary refractory disease defines the majority of treatment failures in our series. Our CAR T-cell experience was limited given the time frame of this study. Desai et al. reported improved outcomes with CAR T-cell in patients with R/R HGBCL-DH/TH, noting increasing time to cellular therapy was associated with inferior OS [15]. However, this study was largely driven by advanced-stage patients (82%).

The role of CNS prophylaxis is currently controversial. However, historically, HGBCL-DH/TH are associated with high-rates of CNS relapse. In our series, although only one-third of patients received CNS prophylaxis, no CNS relapses occurred, suggesting CNS prophylaxis may be safely omitted for most LS HGBCL-DH/TH. Only 2% of the patients were characterized by a high CNS-IPI score, which, although imperfect for predicting the risk of CNS relapse, is routinely used to guide CNS prophylaxis therapy. It is possible that the CNS relapse risk that we observe with HGBCL-DH/TH is associated with disease burden and high-risk extranodal compartments. The role of CNS prophylaxis in LS HGBCL-DH/TH warrants further attention, and if our findings are validated, it may lead to our ability to forgo CNS prophylaxis for patients with LS HGBCL-DH/TH.

Despite the fact that prior publications have demonstrated the relevance of *MYC* partner in HGBCL-DH/TH, we were not able to replicate these findings. It is highly possible that this assessment was limited by the small number of archival tissue available. Limitations of study include retrospective nature of this study, as well as relatively small population. Given the small sample size, multivariate analysis could not be performed. However, this is one of the largest dedicated studies evaluating LS HGBCL-DH/TH and adds valuable information to this small field. Multicenter endeavors to further evaluate this unique population, including *MYC* partner analysis, is warranted especially in the CAR T-cell era.

## Data Availability

The datasets generated during and/or analyzed during the current study are available from the corresponding author on reasonable request.

## References

[1] Petrich AM, Gandhi M, Jovanovic B, Castillo JJ, Rajguru S, Yang DT, et al. Impact of induction regimen and stem cell transplantation on outcomes in double-hit lymphoma: a multicenter retrospective analysis. Blood. 2014;124:2354–61. 25161267 10.1182/blood-2014-05-578963

[2] Landsburg DJ, Falkiewicz MK, Maly J, Blum KA, Howlett C, Feldman T, et al. Outcomes of Patients With Double-Hit Lymphoma Who Achieve First Complete Remission. J Clin Oncol : Off J Am Soc Clin Oncol. 2017;35:2260–7. Jco2017722157 10.1200/JCO.2017.72.2157 PMC6366297 28475457

[3] Copie-Bergman C, Cuillière-Dartigues P, Baia M, Briere J, Delarue R, Canioni D, et al. MYC-IG rearrangements are negative predictors of survival in DLBCL patients treated with immunochemotherapy: a GELA/LYSA study. Blood. 2015;126:2466–74. 26373676 10.1182/blood-2015-05-647602

[4] Clipson A, Barrans S, Zeng N, Crouch S, Grigoropoulos NF, Liu H, et al. The prognosis of MYC translocation positive diffuse large B-cell lymphoma depends on the second hit. J Pathol Clin Res. 2015;1:125–33. 27347428 10.1002/cjp2.10 PMC4915334

[5] Rosenwald A, Bens S, Advani R, Barrans S, Copie-Bergman C, Elsensohn M-H, et al. Prognostic Significance of MYC Rearrangement and Translocation Partner in Diffuse Large B-Cell Lymphoma: A Study by the Lunenburg Lymphoma Biomarker Consortium. J Clin Oncol. 2019;37:3359–68. 31498031 10.1200/JCO.19.00743

[6] Alduaij W, Collinge B, Ben-Neriah S, Jiang A, Hilton LK, Boyle M, et al. Molecular determinants of clinical outcomes in a real-world diffuse large B-cell lymphoma population. Blood. 2023;141:2493–507. 36302166 10.1182/blood.2022018248

[7] Campo E, Jaffe ES, Cook JR, Quintanilla-Martinez L, Swerdlow SH, Anderson KC, et al. The International Consensus Classification of Mature Lymphoid Neoplasms: a report from the Clinical Advisory Committee. Blood. 2022;140:1229–53. 35653592 10.1182/blood.2022015851 PMC9479027

[8] Alaggio R, Amador C, Anagnostopoulos I, Attygalle AD, Araujo IBDO, Berti E, et al. The 5th edition of the World Health Organization Classification of Haematolymphoid Tumours: Lymphoid Neoplasms. Leukemia. 2022;36:1720–48. 35732829 10.1038/s41375-022-01620-2 PMC9214472

[9] Wright GW, Huang DW, Phelan JD, Coulibaly ZA, Roulland S, Young RM, et al. A Probabilistic Classification Tool for Genetic Subtypes of Diffuse Large B Cell Lymphoma with Therapeutic Implications. Cancer cell. 2020;37:551–68.e14. 32289277 10.1016/j.ccell.2020.03.015 PMC8459709

[10] Chapuy B, Stewart C, Dunford AJ, Kim J, Kamburov A, Redd RA, et al. Molecular subtypes of diffuse large B cell lymphoma are associated with distinct pathogenic mechanisms and outcomes. Nat Med. 2018;24:679–90. 29713087 10.1038/s41591-018-0016-8 PMC6613387

[11] Künstner A, Witte HM, Riedl J, Bernard V, Stölting S, Merz H, et al. Mutational landscape of high-grade B-cell lymphoma with MYC-, BCL2 and/or BCL6 rearrangements characterized by whole-exome sequencing. Haematologica. 2022;107:1850–63. 34788985 10.3324/haematol.2021.279631 PMC9335106

[12] Torka P, Kothari SK, Sundaram S, Li S, Medeiros LJ, Ayers EC, et al. Outcomes of patients with limited-stage aggressive large B-cell lymphoma with high-risk cytogenetics. Blood Adv. 2020;4:253–62. 31945157 10.1182/bloodadvances.2019000875 PMC6988401

[13] Tilly H, Morschhauser F, Sehn LH, Friedberg JW, Trněný M, Sharman JP, et al. Polatuzumab Vedotin in Previously Untreated Diffuse Large B-Cell Lymphoma. N. Engl J Med. 2022;386:351–63. 34904799 10.1056/NEJMoa2115304 PMC11702892

[14] Morschhauser F, Leung W, Raghavan V, Lenz G, Jardin F, Herrera AF, et al. Deciphering the Clinical Benefit of Pola-R-CHP versus R-CHOP in Different Genetic Subtypes Beyond Cell of Origin in the POLARIX Study. Blood. 2023;142:3000.

[15] Desai SH, Sumransub N, Evans R, Watkins MP, Karmali R, Goyal G, et al. Improved Survival of R/R Double Hit/Triple Hit Lymphoma in the Era of CD19 Chimeric Antigen T Cell (CART) Therapy. Blood. 2023;142:308.37498584

